# Facile fabrication of amino-functionalized MIL-68(Al) metal–organic framework for effective adsorption of arsenate (As(V))

**DOI:** 10.1038/s41598-022-16038-0

**Published:** 2022-07-13

**Authors:** Alireza Rahmani, Amir Shabanloo, Solmaz Zabihollahi, Mehdi Salari, Mostafa Leili, Mohammad Khazaei, Saber Alizadeh, Davood Nematollahi

**Affiliations:** 1 Department of Environmental Health Engineering, Faculty of Health and Research Center for Health Sciences, Hamadan University of Medical Sciences, Hamadan, Iran; 2 Department of Environmental Health Engineering, Hamadan University of Medical Sciences, Hamadan, Iran; 3 Faculty of Chemistry, Bu-Ali-Sina University, Hamadan, Iran

**Keywords:** Environmental sciences, Environmental chemistry

## Abstract

An amino-functionalized MIL-68(Al) metal–organic framework (amino-MIL-68(Al) MOF) was synthesized by solvothermal method and then characterized by FESEM, XRD, FTIR, EDX-mapping, and BET-BJH techniques. In order to predict arsenate (As(V)) removal, a robust quadratic model (R^2^ > 0.99, *F*-value = 2389.17 and *p* value < 0.0001) was developed by the central composite design (CCD) method and then the genetic algorithm (GA) was utilized to optimize the system response and four independent variables. The results showed that As(V) adsorption on MOF was affected by solution pH, adsorbent dose, As(V) concentration and reaction time, respectively. Predicted and experimental As(V) removal efficiencies under optimal conditions were 99.45 and 99.87%, respectively. The fitting of experimental data showed that As(V) adsorption on MOF is well described by the nonlinear form of the Langmuir isotherm and pseudo-second-order kinetic. At optimum pH 3, the maximum As(V) adsorption capacity was 74.29 mg/g. Thermodynamic studies in the temperature range of 25 to 50 °C showed that As(V) adsorption is a spontaneous endothermic process. The reusability of MOF in ten adsorption/regeneration cycles was studied and the results showed high reusability of this adsorbent. The highest interventional effect in inhibiting As(V) adsorption was related to phosphate anion. The results of this study showed that amino-MIL-68(Al) can be used as an effective MOF with a high surface area (> 1000 m^2^/g) and high reusability for As(V)-contaminated water.

## Introduction

Contamination of water resources with arsenic (As) is a major environmental threat because As, in addition to acute toxicity and high mobility in water sources, has a high accumulation capacity in the food chain and aquatic organisms, so it can cause serious diseases such as skin, kidney, liver and lung cancers in humans^1^. As is recognized by the World Health Organization (WHO) as a priority issue and the guideline for its concentration in drinking water is 10 µg/L^2,3^. The toxicity of As depends on the species and its oxidation state. Inorganic species of As in the aquatic environment include arsenite (As (III)) and arsenate (As (V)). In alkaline water sources (pH > 7.5) the predominant species is As(III), which is 60 times more toxic than As(V)^4^. Organic As species including dimethyl arsenic acid, monomethylarsonic acid and arsenobetaine are about 70 times less toxic than inorganic As species^5^. Various processes have been studied to remove As from contaminated water, including nanofiltration^6^, electrochemical techniques^7^, chemical precipitation^8^, ion exchange^9^, and membrane separation^10^. Production of excess sludge, high cost and energy requirement, incomplete removal of pollutants, high chemical requirements and high costs of operation and maintenance are disadvantages of previous methods^11^.

Adsorption process is an easy, efficient, cost-effective and environmentally friendly method that is widely used to remove toxic elements from contaminated water^12^. However, the performance of commercial adsorbents such as activated carbon, activated alumina and powdered zeolite for As adsorption has not been satisfactory^13^. Therefore, the main focus of research is on the development of a new group of porous adsorbents that, despite their high reusability, are able to increase the adsorption capacity and adsorption kinetics of As^14^.

In this regard, in the last decade, promising and emerging adsorbents called Metal Organic Frameworks (MOFs) have received special attention. MOFs are a unique group of crystalline porous materials whose skeleton consists of coordinated bonds of metal nodes and organic linkers^15^. Hence, they are also referred to as porous coordination polymers (PCPs)^16^. Large surface area (1000 to 10,000 m^2^/g), high porosity and high crystallinity are the most important attractive properties of MOFs^14^. Thus, different types of MOFs have been successfully developed to adsorb environmental pollutants^17–20^. Early MOFs could not be used in water due to the instability of the ligand–metal bonds because they decomposed gradually when exposed to moisture. However, metal carboxylate frameworks containing high-valence metal ions are in the group of water-stable MOFs^21^. The MIL family is a group of MOFs composed of trivalent metal cations such as Al^3+^, Cr^3+^, V^3+^, In^3+^ and Ga^3+^ and carboxylic acid groups^22^. Among these, Al^3+^ based MOFs are known as stable adsorbents for adsorption of water pollutants^23^.

One of the best-known MOFs in the MIL family is MIL-68(Al), in which the octahedral structures of AlO_4_(OH)_2_ are linked together by hydroxyl groups and terephthalate ligands^24^. According to Tehrani et al., the maximum adsorption capacity of Methylene Blue and Rhodamine B dyes on MIL-68(Al) was 1666 and 1111 mg/g, respectively^25^. However, the practical application of most MOFs in industrial conditions is limited due to their poor chemical stability^26^. Research has shown that functionalization of MOFs with amine groups can significantly increase their chemical stability^27^. In addition, modification of MOFs with amine groups increases the number of electron-rich nitrogen sites, which results in more positive charge in the MOF structure^28^. Some MOFs functionalized with amine groups such as UiO-66-NH_2_^29^, NH_2_-Cu-MOF^30^, amino-functionalized MOF-5^31^ have recently been studied to remove various water contaminants.

However, little attention has been paid to the application of MIL-68(Al) functionalized with—amine groups as a water-stable MOF for the removal of heavy metals from contaminated water. Therefore, the aim of this study was to synthesize MIL-68(Al) functionalized with amine group (–NH_2_) (amino-MIL-68(Al)) and use it to remove As(V) from water. Modeling and optimization of the effect of four independent variables including solution pH, MOF dose, reaction time and As(V) concentration on the adsorption process were performed using response surface methodology-central composite design (RSM-CCD) and genetic algorithm (GA), respectively. Under optimal conditions of adsorption kinetics and isotherms and the effect of interfering anions on the adsorption process was investigated.

## Materials and methods

### Materials

All of the reagents including 2-aminoterephthalic acid (NH_2_-H_2_BDC), aluminum chloride hexahydrate (AlCl_3_. 6H_2_O), N, N-Dimethylformamide (DMF), and methanol (MeOH) were purchased from Merck Company and utilized without any purification. Disodium hydrogen arsenate (Na_2_HAsO_4_.7H_2_O), hydrochloric acid (HCl), sodium hydroxide (NaOH) was also purchased from Merck Company.

### Synthesis amino-MIL-68(Al)

Preparation of MIL-68(Al) by the solvothermal method has been reported in previous studies^24,32^. In the present study, 2-aminoterephthalic acid was used as a ligand for the synthesis of amino-MIL-68(Al) instead of terephthalic acid. As briefly, the synthesis was accomplished by dissolving 5.0 g (30 mmol) 2-aminoterephthalic acid (NH_2−_H_2_BDC) and 4.88 g (20 mmol) AlCl_3_·6H_2_O in 300 mL of DMF. The prepared precursor was transferred into a round flask and kept under reflux for 18 h at 130 °C. After cooling down of mixture, the solid was centrifuged and rinsed three times by DMF for remove the unreacted ligands. Also, the obtained solid was dispersed in MeOH and rinsed for Three times for remove of the DMF. Finally, the amino-MIL-68(Al) MOF was aged overnight at 100 °C.

### Characterization of amino-MIL-68(Al)

The morphology of the synthesized MOF surface was studied with field emission scanning electron microscopy (FESEM) images (FEI-Nova NanoSEM 450). The crystalline structure of the MOF was investigated by X-ray diffraction (XRD) analysis (Ultima IV, Rigaku). Functional groups in the MOF structure were also determined by Fourier transform infrared spectroscopy (FTIR, Perkin-Elmer, spectrum 65, Waltham, USA). Energy-dispersive X-ray spectroscopy (EDX) mapping (Bruker XFlash6L10) was used to observe the composition of the elements and their distribution on the synthesized adsorbent surface. Finally, the textural properties of the amino-MIL-68(Al) such as specific surface area and pore size were studied by ADS/DES isotherms of nitrogen obtained at 77 K (BELsorp mini II, BEL, Japan). The Zeta potential of the prepared MOF at pH 3–11 was measured using a Zeta Sizer (Malvern, England).

### CCD experimental design

Experimental design and statistical analysis of data were performed with RSM-CCD. The main advantage of this method is the development of a mathematical model with a small number of experiments that can identify the optimal points of independent variables and evaluate the effect of variables as well as the interaction between them^33,34^. In the present study, the effect of four independent variables including solution pH (A), amino-MIL-68(Al) dose (B), reaction time (C) and As(V) concentration (D) on the efficiency of As(V) removal as a system response (Y) was studied by a five-level full CCD. Table 1 shows four independent variables and their five coded levels. Independent variables and their ranges were selected based on experimental data obtained from the pre-test. The designed experiments are also presented in Table 2. Each experimental run was performed with three replications and their mean response was presented as As(V) removal efficiency in Table 2. Statistical analysis of variance (ANOVA) was used to determine the regression coefficients of the model. The graphical relationship between the variables and the system response was also demonstrated using response surface plots. The optimization of independent variables was also performed with the aim of maximizing the efficiency of As(V) removal by applying the final model equation in the GA tool. Design-Expert v13 (https://www.statease.com/docs/v13/) and MATLAB R2013a (https://www.mathworks.com/products/matlab.html) software were used to perform CCD and GA in this study, respectively.

**Table 1:** Independent variables and their coded levels.

Variable	Symbols	− α	− 1	0	+ 1	+ α
pH	A	3	5	7	9	11
Amino-MIL-68(Al) dosage (g/L)	B	0.05	0.14	0.23	0.31	0.4
Reaction time (min)	C	10	27.5	45	62.5	80
As(V) concentration (mg/L)	D	2.5	14.375	26.25	38.125	50

**Table 2:** Experimental design with As(V) removal efficiency.

Run	Variable 1 A: pH	Variable 2 B: MOF dosage (g/L)	Variable 3 C: Reaction time (min)	Variable 4 D: As(V) concentration (mg/L)	System response Y: As removal (%)
1	5	0.31	27.5	38.125	44.8
2	7	0.23	10	26.25	24.2
3	7	0.4	45	26.25	55.8
4	7	0.23	45	26.25	39.6
5	7	0.23	45	50	26.5
6	5	0.14	27.5	38.125	26.4
7	9	0.31	62.5	14.375	48.1
8	7	0.23	45	26.25	38.5
9	5	0.14	27.5	14.375	40.6
10	7	0.23	45	2.5	54.7
11	9	0.14	27.5	38.125	2.1
12	9	0.14	62.5	14.375	27.6
13	7	0.23	45	26.25	39.6
14	7	0.23	80	26.25	48.3
15	3	0.23	45	26.25	62.4
16	5	0.31	62.5	14.375	71.4
17	9	0.31	62.5	38.125	33.3
18	5	0.31	62.5	38.125	60.2
19	5	0.14	62.5	14.375	52.1
20	5	0.14	62.5	38.125	40.9
21	5	0.31	27.5	14.375	56.3
22	7	0.23	45	26.25	39.5
23	9	0.14	62.5	38.125	11.1
24	11	0.23	45	26.25	13.2
25	7	0.23	45	26.25	39.6
26	9	0.14	27.5	14.375	19.6
27	7	0.23	45	26.25	39.7
28	7	0.05	45	26.25	15.2
29	9	0.31	27.5	14.375	38.6
30	9	0.31	27.5	38.125	22.4

### As(V) adsorption experiments

Adsorption experiments were performed in a batch reactor containing the desired concentrations of As(V) (D: 2.5–50 mg/L) and amino-MIL-68(Al) MOF (B: 0.05–0.4 g/L). The effect of four independent variables defined in Table 1 was studied on the efficiency of As(V) adsorption. As(V) solutions were prepared synthetically by disodium hydrogen arsenate (Na_2_HAsO_4_·7H_2_O). In order to completely mix the sample solution containing As(V) and MOF, a magnetic stirrer with a stirrer speed of 100 rpm was used. 0.1 NaOH and 0.1 HCl solutions were used to adjust the pH (A: 3–11) of the sample solution (SensION, HACH). After a specified time (C: 10–80 min), the samples were centrifuged for 10 min at 7000 rpm to separate the MOF from the solution. As(V) concentrations were determined using ICP-MS before and after the adsorption process^35^. As(V) adsorption efficiency (Y, %) and adsorption capacity (q_e_, mg/g) were calculated by Eqs. (1) and (2). After optimizing the As(V) adsorption on the amino-MIL-68(Al), the reusability of the adsorbent was evaluated for ten consecutive reuses. Also, the effect of interfering anions on As(V) adsorption efficiency in the presence of ${\text{Cl}}^{ - }$, ${\text{SO}}_{4}^{2 - }$, ${\text{NO}}_{3}^{ - }$ and ${\text{PO}}_{4}^{3 - }$ were evaluated.

1 $${\text{Y }}\left( {\text{\% }} \right) = \frac{{{\text{C}}_{0} - {\text{C}}_{{\text{e}}} }}{{{\text{C}}_{0} }} \times 100$$

2 $${\text{q}}_{{\text{e}}} = \frac{{({\text{C}}_{0} - {\text{ C}}_{{\text{e}}} ){\text{V}}}}{{\text{m}}}$$

where C_0_ (mg/L) and C_e_ (mg/L) represent the initial and final concentrations of As(V), respectively, m (mg) is assigned to the mass of amino-MIL-68(Al) MOF, and V (L) is related to the sample solution.

## Results and discussion

### Characterization of amino-MIL-68(Al) and the effect of amine group in improving MOF properties

**Figure 1:**
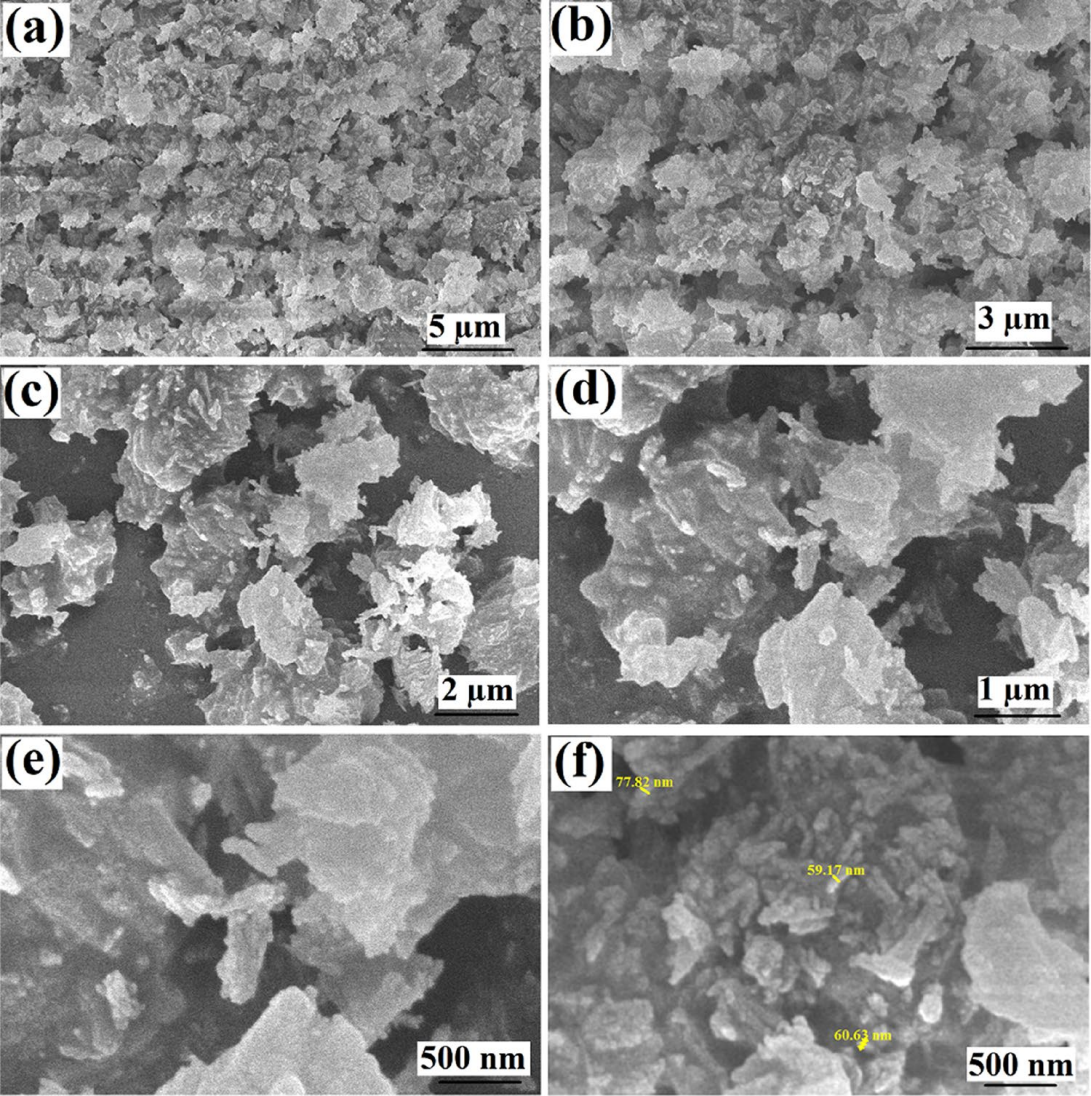
FESEM images of amino-MIL-68(Al) with different magnifications (**a**–**f**).

**Figure 2:**
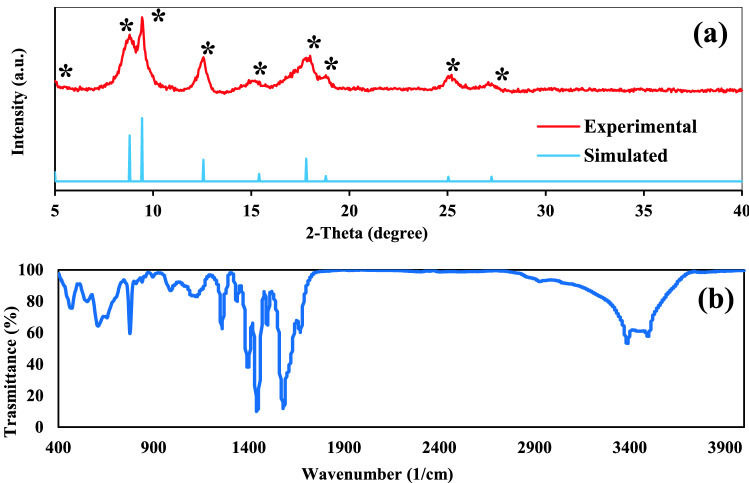
Experimental and simulated XRD patterns of amino-MIL-68(Al) (**a**); FTIR spectra of amino-MIL-68(Al) (**b**).

Figure 1 shows the morphology of the amino-MIL-68(Al) surface at different magnifications. As can be seen, the MOF surface consists of cumulative amorphous particles. According to Fig. 1f, the particle diameters are in the range of 60 to 80 nm. In Wu et al.'s study, a similar structure was reported for aluminum-based MOFs^36^. The XRD spectrum of amino-MIL-68(Al) is presented in Fig. 2a. The Sharp characteristic peaks observed at 2θ = 5, 8.8, 9.44, 12.6, 15.25, 17.8, 18.92, 24.9 and 26.8° have been reported in previous studies, indicating that the MIL-68(Al) is well synthesized in the MOF structure^36–40^. The functional groups of the synthesized amino-MIL-68(Al) can be seen in Fig. 2b. The bands appearing at 990, 1257 and 1337 1/cm belong to the n(C–N) absorption distinctive of aromatic amines. N–H vibration can be seen at bands 1580 and 776 1/cm. The peak at around 1440 1/cm is attributed to the stretching vibration of C=C of 2-aminoterephthalic acid^41^. C–H and C=C of the benzene rings can be identified at bands 1123 and 1395 1/cm, respectively. The primary amines –NH_2_ on organic linkers can be found at band 3386 1/cm. The band at 3495 1/cm belongs to the vibration of OH group. Finally, the bands at 472, 551, and 608 1/cm belong to the vibrations of the metal center of Al–O^42^.

**Figure 3:**
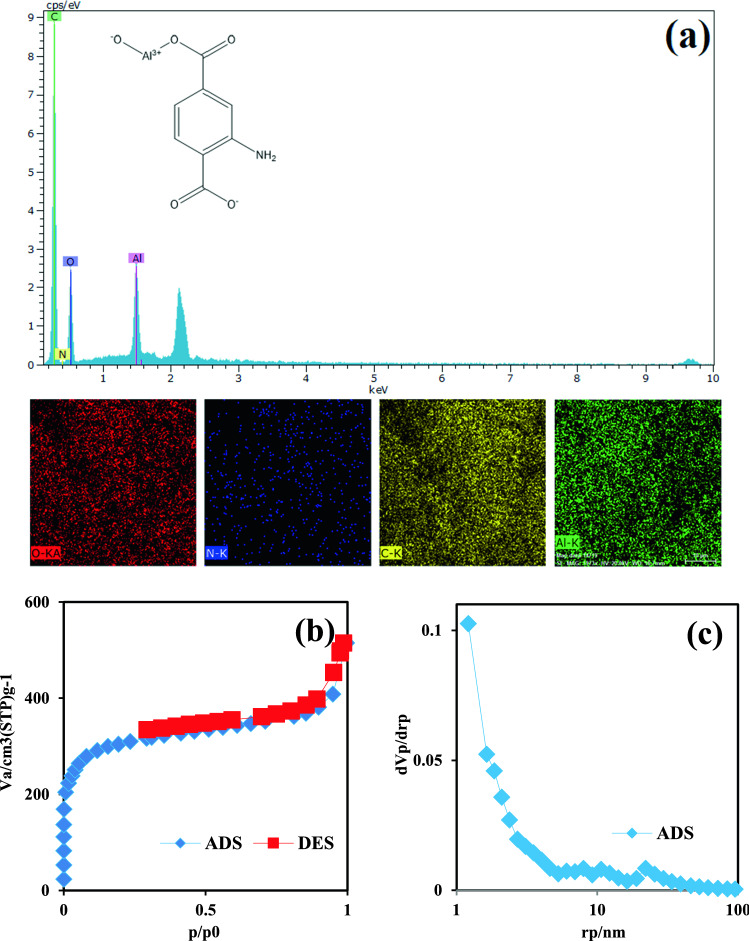
EDX-mapping analysis of amino-MIL-68(Al) (**a**). The ADS/DES isotherm of N_2_ on amino-MIL-68(Al) (**b**), BJH pore size of amino-MIL-68(Al) (**c**).

Figure 3a shows the EDX-mapping analysis of the amino-MIL-68(Al) surface. As can be seen, the synthesized amino-MIL-68(Al) consists of a uniform distribution of the elements carbon (61.81 wt%), oxygen (31.6 wt%), aluminum (4.35 wt.%), and nitrogen (2.25 wt%). The chemical structure of amino-MIL-68(Al) is shown in Fig. 3. Accordingly, its chemical formula and molecular mass are ${\text{C}}_{8} {\text{H}}_{5} {\text{AlNO}}_{5}^{ + }$ and 222.11 g/mol, respectively. In addition, the theoretical analysis of the elements indicates that the mass percentages (wt%) of carbon, oxygen, aluminum, nitrogen and hydrogen are 43.26, 36.02, 12.15, 6.31 and 2.27, respectively. As can be seen, the mass ratio of aluminum to nitrogen is almost double, which is consistent with the EDX results. Figures 3b,c show the ADS/DES isotherm of nitrogen and Barrett-Joyner-Halenda (BJH) pore size of amino-MIL-68(Al), respectively. The shape of the isotherm is similar to type II with H3 type hysteresis, suggesting the presence of mesoporous texture with the micropores^43^. The results also showed that the Brunner−Emmet −Teller (BET) surface area for the synthesized amino-MIL-68(Al) is 1170.9 m^2^/g, which is much larger than the surface area reported for MIL-88B(Fe) (214 m^2^/g)^44^, NH_2_-MIL-88(Fe) (201 m^2^/g)^45^ and NH_2_-MIL-68(In) (655 m^2^/g)^46^. The mean pore diameter and total pore volume for amino-MIL-68(Al) were also 2.64 nm and 0.7743 cm^3^/g, respectively.

**Figure 4:**
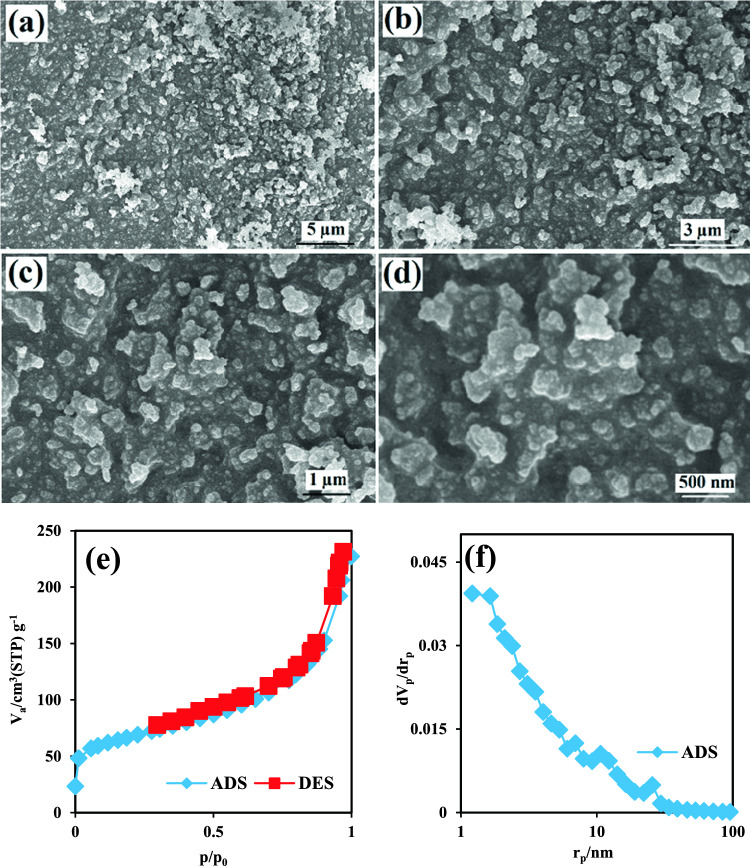
FESEM images of MIL-68(Al) with different magnifications (**a**–**d**); The ADS/DES isotherm of N_2_ on MIL-68(Al) (**e**), BJH pore size of MIL-68(Al) (**f**).

Figures 4a–d show the surface morphology of MIL-68(Al) at different magnifications. As can be seen, the surface morphology of the amino-MIL-68(Al) (Fig. 1) is much less porous compared to MIL-68(Al), which reduces the surface area of the MOF in the absence of the amine group. The results of the ADS/DES isotherm of nitrogen, as well as the BJH pore size of MIL-68(Al), are presented in Fig. 4e,f, respectively. As expected, the BET surface area for MIL-68(Al) is 239.98 m^2^/g, which is less than the amino-MIL-68(Al). In other words, functionalization of MIL-68(Al) with the amine group increases the surface area of MOF by about 5 times, which means increasing the active sites for the absorption of environmental pollutants. In addition, the results showed that the mean pore diameter and total pore volume for MIL-68(Al) were 5.73 nm and 0.3436 cm^3^/g, respectively.

### Data analysis, process modeling and optimization

To model As(V) adsorption on amino-MIL-68(Al), experimental design was performed based on CCD method. Table 2 shows the efficiency of As(V) removal in designed experiments. Data analysis showed that a quadratic model is able to predict system response with R^2^ > 0.99. ANOVA results for As(V) adsorption by amino-MIL-68(Al) are shown in Table 3. For the model, F-value and p-value are 2389.17 and < 0.0001, respectively, which confirms that the developed model is statistically significant. However, Lack of Fit is statistically non-significant because the values of these parameters are 1.23 and 0.4341, respectively. These results indicate that the data fit well with the developed quadratic model^47,48^. The final model equation is presented in Eq. (3). As can be seen, the proposed model is affected by the linear (A, B, C and D), interaction (AB, AC, AD, BC, BD and CD) and quadratic (A^2^, B^2^, C^2^ and D^2^) effects of the independent variables. Equation (4) was used to calculate the percentage effect of each parameter on the system response^34^. The final column in Table 3 shows the percentage of the positive and negative effects of each of the model parameters on the system response. As can be seen, the greatest effects on the system response are related to the linear effects of solution pH (A: − 41.34%), amino-MIL-68(Al) dose (B: + 28.70), As(V) concentration (D: − 14.29%) and reaction time (C: 9.98%), respectively. In addition, the system response is totally 3.89% affected by the interaction effects of the independent variables. Among the quadratic effects, the most important parameter affecting the system response is the amino-MIL-68(Al) dose (B^2^: − 0.178%).

3 $$\begin{aligned} {\text{Y }}\left( {\text{\% }} \right) = & 35.49 - 2.68{\text{A}} + 115.29 + 0.686{\text{C}} - 0.554{\text{D}} \\ & + 3.36{\text{AB}} - 0.034{\text{AC}} - 0.0444{\text{AD}} + 0.333{\text{BC}} \\ & + 0.352{\text{BD}} + 0.0017{\text{CD}} - 0.098{\text{A}}^{2} \\ & - 108.024{\text{B}}^{2} - 0.0025{\text{C}}^{2} + 0.00217{\text{D}}^{2} \\ \end{aligned}$$

4 $${\text{Effect }}\left( {\text{\% }} \right) = \left[ {{\raise0.7ex\hbox{${\beta_{i}^{2} }$} \!\mathord{\left/ {\vphantom {{\beta_{i}^{2} } {{\Sigma }\left( {\beta_{i}^{2} } \right)}}}\right.\kern-\nulldelimiterspace} \!\lower0.7ex\hbox{${{\Sigma }\left( {\beta_{i}^{2} } \right)}$}}} \right] \times 100$$

where Y indicates the system response or As(V) removal efficiency (%). A, B, C and D represent the independent variables defined in Table 1. β_i_ is also the regression coefficients of the parameters in the model equation based on coded factors.

**Table 3:** ANOVA results for As(V) adsorption on amino-MIL-68(Al).

Source	Sum of Squares	df	Mean Square	F-value	*p* value	Effect (%)
Model	7927.32	14	566.24	2389.17	< 0.0001	
A*	3467.68	1	3467.68	14,631.48	< 0.0001	− 41.34553
B	2320.48	1	2320.48	9791.01	< 0.0001	28.7074
C	838.1	1	838.1	3536.29	< 0.0001	9.98696
D	1198.77	1	1198.77	5058.08	< 0.0001	− 14.29214
AB	5.24	1	5.24	22.12	0.0003	0.398128
AC	22.8	1	22.8	96.2	< 0.0001	− 1.626429
AD	17.85	1	17.85	75.32	< 0.0001	− 1.279025
BC	3.94	1	3.94	16.64	0.001	0.297481
BD	2.03	1	2.03	8.58	0.0104	0.154466
CD	2.03	1	2.03	8.57	0.0104	0.144137
A^2^	4.24	1	4.24	17.91	0.0007	− 0.178448
B^2^	18.37	1	18.37	77.5	< 0.0001	− 0.783167
C^2^	16.7	1	16.7	70.46	< 0.0001	− 0.700305
D^2^	2.56	1	2.56	10.81	0.005	− 0.106394
Residual	3.56	15	0.237			
Lack of Fit	2.53	10	0.2527	1.23	0.4341	

*A, B, C and D represent the independent variables defined in Table 1.

**Figure 5:**
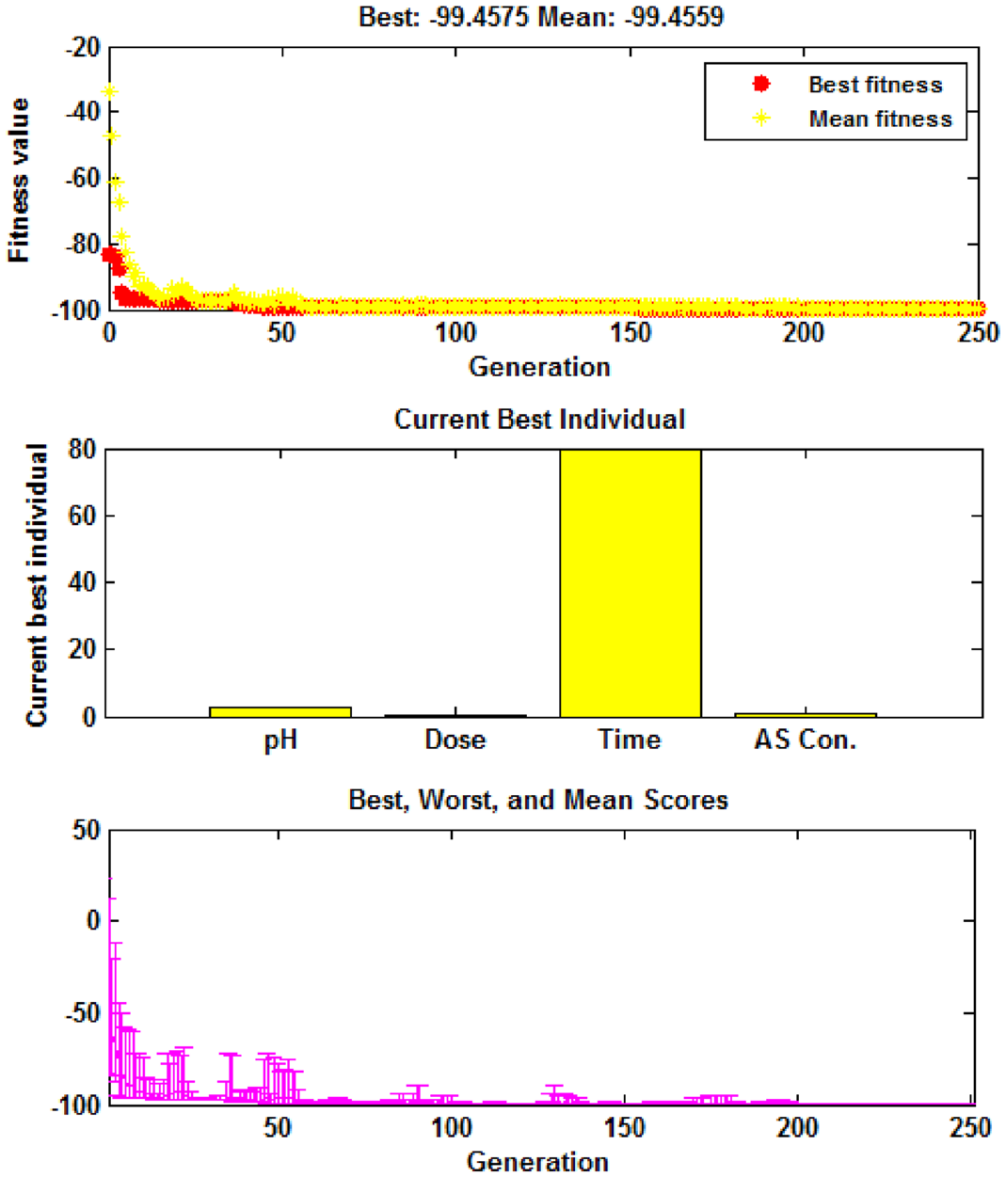
Output of GA method for optimization of independent variables in As(V) adsorption process on amino-MIL-68(Al). MATLAB R2013a software was used to create this figure (https://www.mathworks.com/products/matlab.html).

GA method was utilized to optimize the process and predict the highest As(V) removal efficiency. For this purpose, the equation of the quadratic model was entered into the software as a fitness function, and the independent variables were adjusted to their high and low values (± α)^49^. The software output is shown in Fig. 5. As can be seen, after about 200 generations, the optimal values of the independent variables are predicted. Accordingly, the optimal values for solution pH, amino-MIL-68(Al) dose, reaction time and As(V) concentration were 3, 0.4 (g/L), 80 min and 2.5 mg/L, respectively. For these laboratory conditions, the predicted removal efficiency for As(V) adsorption on amino-MIL-68(Al) was about 99.45%. To evaluate the accuracy of the model, three adsorption experiments were performed under optimal conditions, which showed that the average experimental removal of As(V) (99.87%) is very close to the predicted removal of As(V).

### Interaction of independent variables on the efficiency of As(V) removal

**Figure 6:**
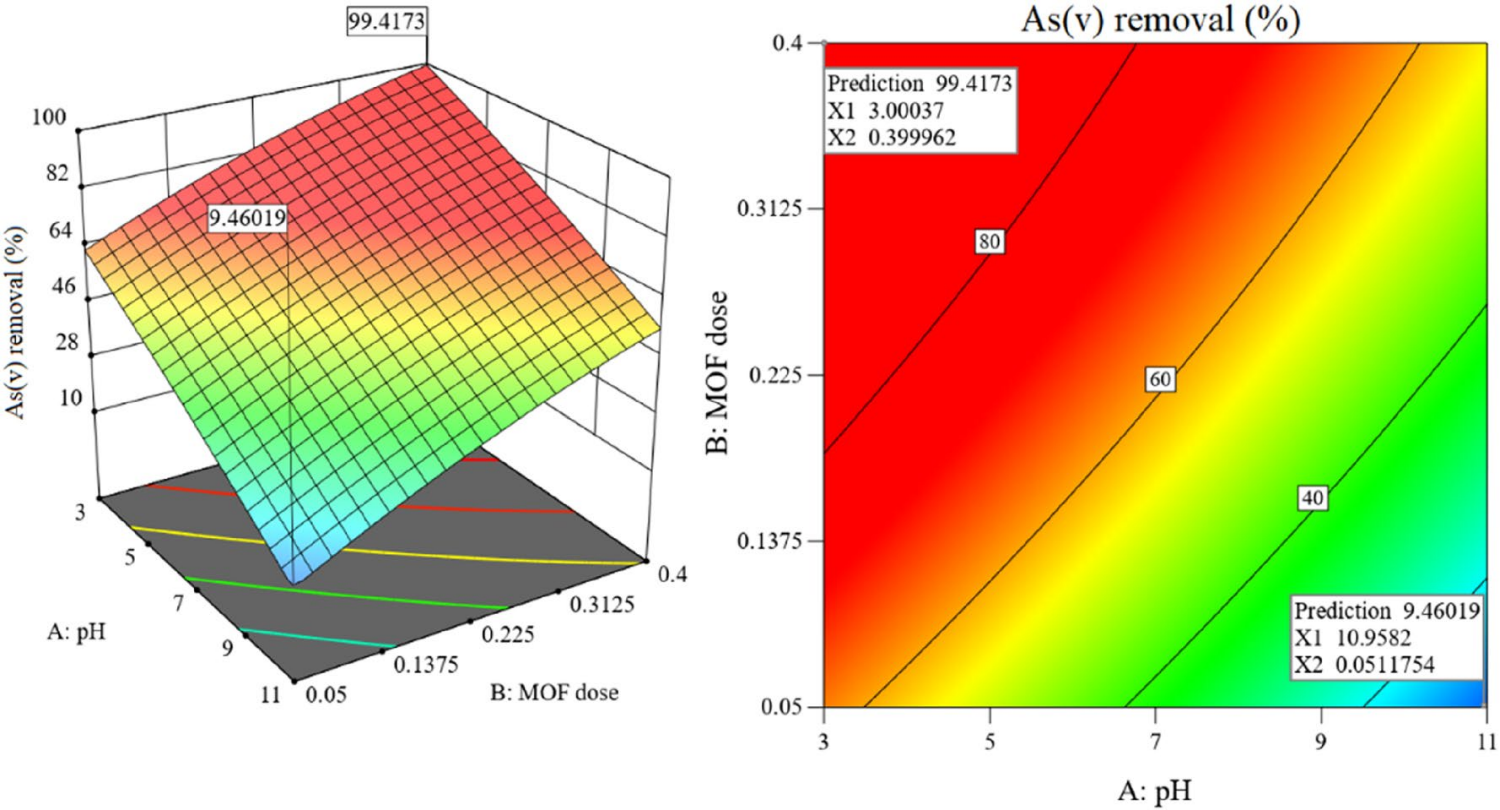
Interaction of pH and MOF dose on system response (As(V) = 2.5 mg/L, Time = 80 min). Design-Expert v13 software was used to create this figure (https://www.statease.com/docs/v13/).

The effect of the interaction of solution pH and MOF dose on As(V) removal efficiency is shown in Fig. 6. Clearly, with decreasing pH in the range of 3 to 11 and with increasing MOF dose in the range of 0.05 to 0.4 g/L, the As(V) removal efficiency is significantly improved. So that at solution pH of 11 and MOF dose of 0.05 g/L, the As(V) removal efficiency is about 9.5%. However, at a solution pH of 3 and in the presence of 0.4 g/L MOF the efficiency of As(V) removal by the model is predicted to be about 99.5%. In addition, as can be seen in the presence of 1 g/L of MOF, the efficiency of As(V) removal at pHs of 5, 7, 9 and 11 is 88.4, 76.9, 68.2 and 54.2%, respectively.

The pH of the solution is one of the operational factors that affects the efficiency of the adsorption process by affecting the properties of the adsorbent surface and the distribution of the dominant species of As(V)^50^. As(V) is mainly present in the form of ${\text{H}}_{2} {\text{AsO}}_{4}^{ - }$ in aqueous solutions with a pH in the range of 3–6. However, with increasing pH (pH > 7), the predominant forms will be ${\text{HAsO}}_{4}^{2 - }$ and ${\text{AsO}}_{4}^{3 - }$^51^. The zeta potential of amino-MIL-68(Al) at pHs of 3, 5, 7, 9, and 11 was measured to be + 11.8, + 9.5, + 4.6, + 1.2, and − 2.3 mV, respectively. Accordingly, the pHzpc for the amino-MIL-68(Al) was determined at 9.2. In other words, in a sample solution with a pH greater than 9.2, the amino-MIL-68(Al) surface charge has a negative state. Accordingly, the amino-MIL-68(Al) efficiency for adsorption of As(V) anionic species at high pH (pH > 9.2) is limited. However, the decrement in As(V) removal efficiency with increasing solution pH in the range of 3 to 9 can be related to the decrease of surface potential of amino-MIL-68(Al), which reduces the electrostatic attraction between the As(V) anions and the MOF surface^52^. Accordingly, the optimal pH in the present study was predicted to be 3, which is consistent with the results of some previous studies. Vu et al. have studied the effect of pH in the range of 3 to 11 on the removal efficiency of As(V) with MIL-53(Fe). In their study, the most As(V) removal was reported at pH 5, 3, 9, and 11, respectively^53^. In the study of Wang et al., the highest As(V) adsorption on UiO-66 was obtained at pH between 1 and 3^13^. In addition, in the study of Wu et al., The highest As(V) removal efficiency on MIL-88A microrods was observed at pH 3 and 5^54^. As can be seen in Fig. 6, at a solution pH of 3, the As(V) removal efficiency in the presence of MOF doses of 0.05, 0.1375, 0.225, 0.3125 and 0.4 g/L is about 61.2, 74.5, 83, 92.1 and 99.5%, respectively. Clearly, as the adsorbent dose increases, the number of available adsorption sites increases, resulting in improved removal efficiencies^55^. Improvement of contaminant removal efficiency by increasing the adsorbent dose has been reported by other scholars^50,56^.

**Figure 7:**
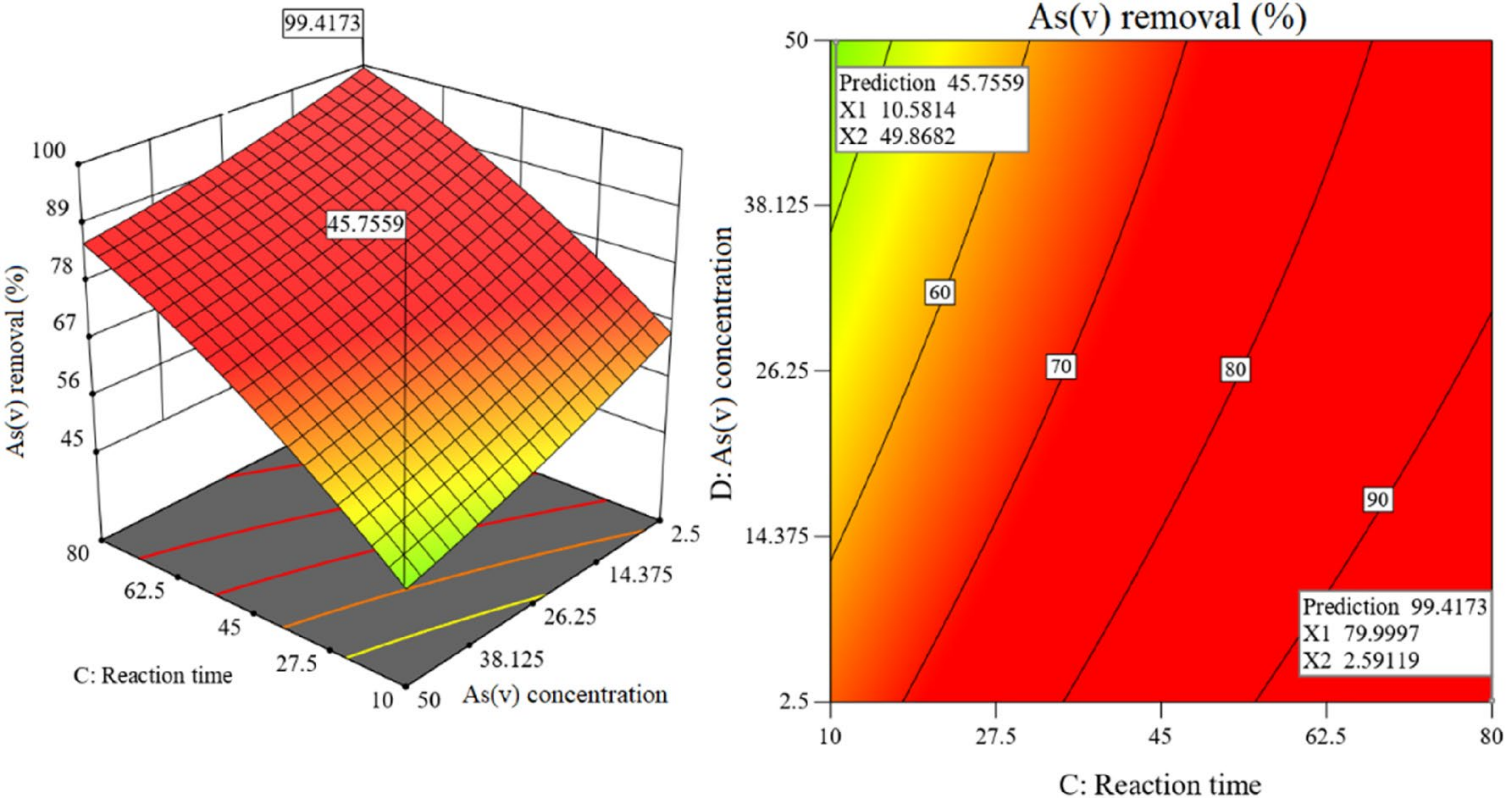
Interaction of reaction time and As(V) concentration on system response (pH = 3, MOF dose = 0.4 g/L). Design-Expert v13 software was used to create this figure (https://www.statease.com/docs/v13/).

The interaction of reaction time and As(V) concentration on As(V) removal efficiency is shown in Fig. 7. As can be seen, increasing the reaction time and decreasing the As(V) concentration improve the removal efficiency. Accordingly, the efficiency of As(V) removal at the initial concentration of 50 mg/L after 10 and 80 min of the reaction is about 45.7 and 85.2%, respectively. On the other hand, the removal efficiency of As(V) at initial concentrations of 2.5 and 50 mg/L after 80 min of reaction is about 99.5 and 85.2%, respectively. At a constant dose of adsorbent, the removal efficiency at high concentrations of As(V) is reduced due to the limited active adsorption sites at the surface of MOF. In this regard, the results reported by other researchers are consistent with our study^50,54^.

### Adsorption isotherms and kinetics

The study of adsorption isotherms can reveal valuable information about the adsorption capacity and behavior of an adsorbent in interaction with an adsorbate^57^. For this purpose, under optimal conditions, the experimental data were evaluated with Freundlich, Langmuir, Temkin and Dublin–Radushkevich isotherm models. The Langmuir isotherm describes the monolayer adsorption on the homogeneous surface of the adsorbent. The Freundlich isotherm assumes that adsorption is not monolayer and describes equilibrium at heterogeneous surfaces^58^. The nonlinear and linear models of the Langmuir isotherm are presented in Eqs. (5 and (6). For Freundlich isotherm, nonlinear and linear models can be seen in Eqs. (7) and (8).

5 $${\text{q}}_{{\text{e}}} = \frac{{{\text{q}}_{{{\text{max}}}} {\text{K}}_{{\text{L}}} {\text{C}}_{{\text{e}}} }}{{1 + {\text{ K}}_{{\text{L}}} {\text{C}}_{{\text{e}}} }}$$

6 $$\frac{{{\text{Ce}}}}{{{\text{qe}}}}{ = }\frac{{1}}{{{\text{ K}}_{{\text{L}}} {\text{q}}_{{{\text{max}}}} { }}}{ + }\frac{{{\text{Ce}}}}{{{\text{q}}_{{{\text{max}}}} { }}}$$

7 $${\text{q}}_{{\text{e}}} = {\text{K}}_{{\text{f}}} {\text{ C}}_{{\text{e}}}^{{\frac{1}{{\text{n}}}}}$$

8 $${\text{Loq q}}_{{\text{e}}} = {\text{log K}}_{{\text{F}}} + \frac{1}{{\text{n}}}$$

where qe is the mg of As(V) adsorbed per g of amino-MIL-68(Al) (mg/g). C_e_ indicates the equilibrium concentration of As(V) (mg/L). q_max_ represents the maximum adsorption capacity (mg/g) and K_L_ represents the Langmuir equilibrium constant (l/mg). K_f_ and 1/n show the adsorption capacity (l/mg) and adsorption intensity, respectively. K_f_ and n are determined from the nonlinear graph qe versus Ce and the linear graph log q_e_ versus log C_e_.

The Temkin isotherm describes the process on a heterogeneous surface with adsorption sites with the same bond energy. Equations (9) and (10) describe the nonlinear and linear models of this isotherm^31^. The Dublin–Radushkevich isotherm describes the adsorption process on the heterogeneous surfaces. However, unlike the Freundlich isotherm, the absorption energy dissipation in this isotherm is linear^59^. The nonlinear and linear models of the Dublin–Radushkevich isotherm are presented in Eqs. (11) and (12).

9 $${\text{q}}_{{\text{e}}} = \frac{{{\text{RT}}}}{{\text{B}}}{\text{ln K}}_{{\text{T}}} {\text{C}}_{{\text{e}}}$$

10 $${\text{q}}_{{\text{e}}} = {\text{B ln K }}_{{\text{T}}} + {\text{B lnC}}_{{\text{e}}}$$

11 $${\text{q}}_{{\text{e}}} = {\text{q}}_{{{\text{max}}}} {\text{ exp}}^{{\beta \varepsilon^{2} }}$$

12 $${\text{ln q }}_{{\text{e}}} = {\text{ln q}}_{{{\text{max}}}} - {\upbeta }\varepsilon^{2} { }$$

13 $${\varepsilon } = {\text{RT Ln}}\left( {1 + \frac{1}{{{\text{C}}_{{\text{e}}} }}} \right)$$

14 $${\text{E}} = \frac{1}{{\sqrt {2{\upbeta }} }}$$

where B represents the Temkin isotherm constant (J/mol). K_T_ is the maximum bond energy (l/mg). R and T are also related to gas constant (8.314 J/K mol) and temperature (K), respectively. q_max_ is the monolayer adsorption capacity in the Dublin–Radushkevich isotherm (mg/g). β also represents the adsorption energy constant in this isotherm. ε is the Polanyi potential calculated by Eq. (13). In the Dublin–Radushkevich isotherm, the most probable free adsorption energy (E, J/mol) is calculated by Eq. (14). E < 8 and 8 < E < 16 kJ/mol show a physical nature and chemical nature, respectively.

**Figure 8:**
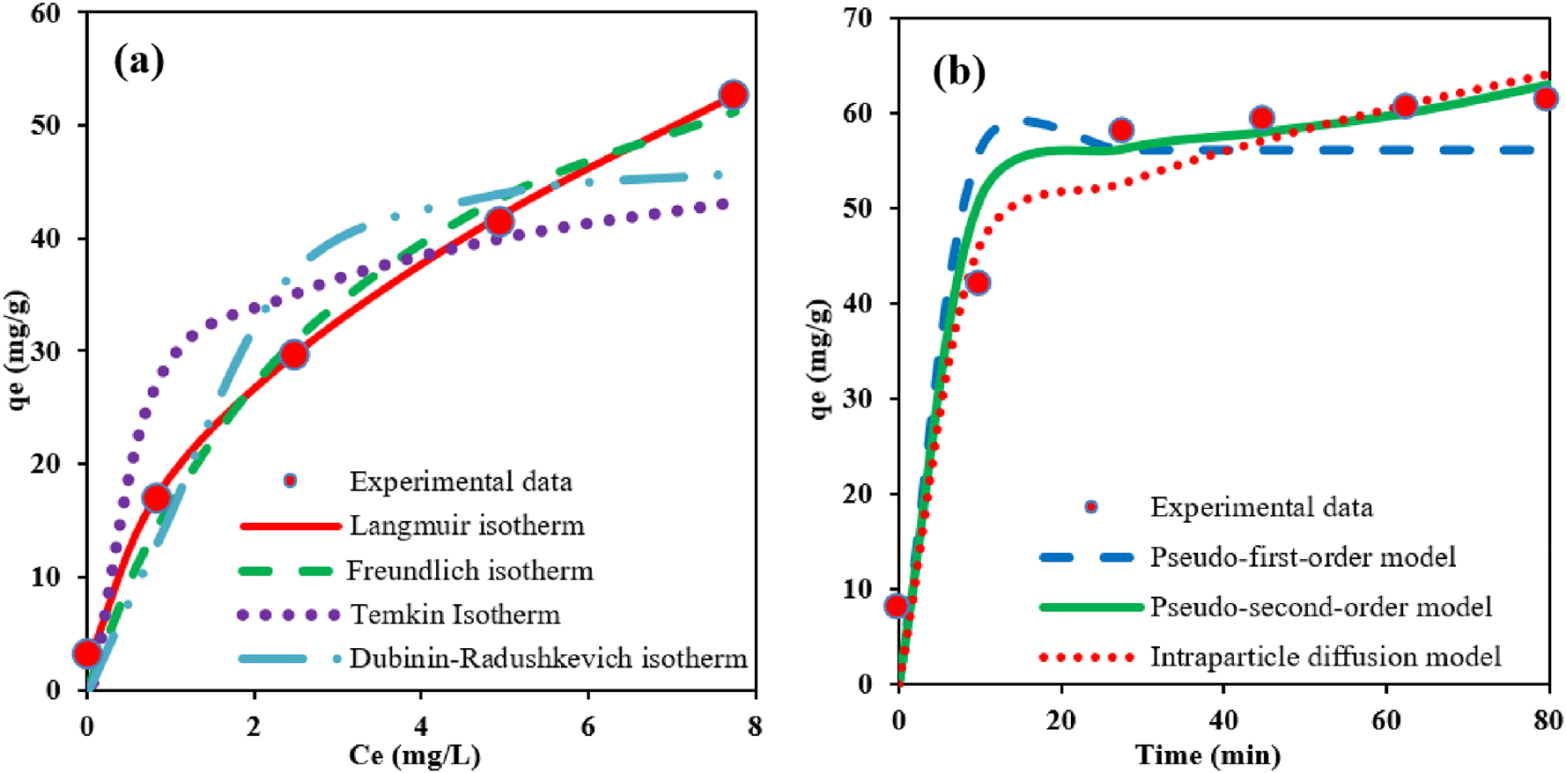
Nonlinear isotherm models (pH = 3) (**a**), and nonlinear adsorption kinetic models (pH = 3, As(V) = 50 mg/L) (**b**) for As(V) adsorption on amino-MIL-68(Al).

The nonlinear form of isotherm models is plotted in Fig. 8a. Table 4 also presents the values of different parameters and coefficients for the studied models. The results show that the Langmuir isotherm (R^2^ = 0.9998) describes the experimental data better than other models. Accordingly, the adsorption of As(V) on amino-MIL-68(Al) is homogeneous monolayer process^14,60^. The maximum adsorption capacity of As(V) by the Langmuir isotherm was obtained to be 74.29 mg/g, which is higher than the reported values for ZrO_2_-sawdust (12 mg/g)^61^, organic biochar (16.2 mg/g)^62^, CuO nanoparticles (22.6 mg/g)^63^, and Fe_3_O_4_-RGO-MnO_2_ (12.22 mg/g)^64^. In addition, the maximum adsorption capacity of As(V) on the synthesized amino-MIL-68(Al) compared to other MOFs including MIL-53(Fe) (21.27 mg/g)^53^, MIL-100(Fe) (110 mg/g)^65^, Fe-BTC (12.29 mg/g)^66^, Fe_3_O_4_@MIL-101(Cr) (80 mg/g)^67^, ZIF-8 (90.92 mg/g)^68^, Co-MOF (96.1 mg/g)^69^, Fe_3_O_4_@UiO-66 (73.2 mg/g)^70^, MOF-808 (24.8 mg/g)^35^, UiO-66 (68 mg/g)^60^ and UiO-66-(SH)2 (10 mg/g)^71^ is an acceptable value.

**Table 4:** Isotherm coefficients and parameters of As(V) adsorption on amino-MIL-68(Al).

Adsorption isotherms	Parameters	Parameter values
Freundlich	K_F_ (mg/g)	18.731
	1/n	0.504
	R^2^	0.9921
Langmuir	q_max_ (mg/g)	74.29
	K_L_ (L/mg)	0.286
	R^2^	0.9998
Temkin	K_T_ (L/g)	135.878
	b (J/mol)	2166.189
	R2	0.8355
Dublin–Radushkevich	q_max_ (mg/g)	47.166
	β (mol^2^/kJ^2^)	0.0286
	R^2^	0.9471
	E	4.181

Determining the reaction rate and its mechanism depends on conducting kinetic studies. Different types of models have been developed to describe the kinetics of adsorption process. In the present study, the adsorption kinetic of As(V) on amino-MIL-68(Al) were studied and fitted with three different kinetic models including pseudo-first order^72^, pseudo-second order^73^, and intraparticle diffusion models^74^. The nonlinear equations of these kinetic models are shown in Eqs. (15) to (17), respectively.

15 $${\text{q}}_{{\text{t}}} = {\text{q}}_{{\text{e }}} \left( {1 - {\text{e}}^{{ - {\text{k}}_{1} {\text{t}}}} } \right)$$

16 $${\text{q}}_{{\text{t}}} = \frac{{{\text{k}}_{2} {\text{q}}_{{\text{e}}}^{2} {\text{t}}}}{{1 + {\text{ k}}_{2} {\text{q}}_{{\text{e}}} {\text{t}}}}$$

17 $${\text{q}}_{{\text{t}}} = {\text{k}}_{{\text{i}}} {\text{t }}^{0/5} + {\text{C}}$$

where q_t_ (mg/g) indicates the absorption capacity at time t. q_e_ (mg/g) is also related to the absorption capacity at equilibrium time. k_1_ (1/min) and k_2_ (g/mg.min) are the rates constant of adsorption for pseudo-first-order and second-first-order kinetic models, respectively. k_i_ (mg/g.min^0/5^) expresses the rate constant of intraparticle diffusion kinetic model.

Figure 8b and Table 5 show the data obtained from the study of adsorption kinetic in the nonlinear form of the models. As can be seen, the experimental data are well consistent with the pseudo-second-order nonlinear model (R^2^ = 0.9822), which indicates that the chemical adsorption mechanism dominates the adsorption process^60^. The results of recent studies show that As(V) adsorption on MOFs (Fe/Mg-MIL-88B(n)^75^, Fe–Co MOF-74^52^, Fe/Al-BDC-NH_2_^76^ and UiO-66/PAN membrane^77^) is generally well described by pseudo-second-order kinetic model.

**Table 5:** Kinetic parameters of As(V) adsorption on amino-MIL-68(Al).

Adsorption kinetics	Parameters	Parameter values
Pseudo-first order model	qe (mg/g)	56.213
	K_1_(min^−1^)	0.413
	R^2^	0.9584
Pseudo-second order model	qe (mg/g)	58.174
	K_2_ (g mg^−1^ min^−1^)	0.221
	R^2^	0.9822
Intraparticle diffusion model	C	36.303
	K_i_ (mg/gmin^0.5^)	0.742
	R^2^	0.9796

### Adsorption thermodynamic

Thermodynamic parameters provide useful information about whether the reactions are endothermic or exothermic, whether the processes are spontaneous or not, and the entropy changes in the process. Thermodynamic parameters were determined using Eqs. (18) to (20)^78^.

18 $${\Delta G}^{^\circ } = - {\text{RT lnK}}_{{\text{c}}}$$

19 $$\ln {\text{K}}_{{\text{c}}} = \frac{{{\Delta S}^{^\circ } }}{{\text{R}}} - \frac{{{\Delta H}^{^\circ } }}{{{\text{RT}}}}$$

20 $${\text{K}}_{{\text{c}}} = \frac{{{\text{q}}_{{\text{e}}} }}{{{\text{C}}_{{\text{e}}} }}$$

where ΔG° is the standard Gibbs free energy (kJ/mol), K_c_ is the distribution coefficient, which was calculated by Eq. (20). T also represents the absolute temperature of the solution (K). ΔS° (J/mol. K) and ΔH° (kJ/mol) are the entropy and enthalpy parameters. In Eq. (20), q_e_ and C_e_ represent the adsorption capacity (mg/g) and As(V) concentration (mg/L) in equilibrium, respectively. The thermodynamic parameters of As(V) adsorption on amino-MIL-68(Al) are presented in Table 6. As can be seen, the value of ΔG° becomes more negative with increasing temperature in the range of 25 to 50 °C. Accordingly, As(V) adsorption on prepared MOF is a spontaneous process that is improved by higher temperatures^76^. Given the positive value of ΔH°, it is clear that the nature of the As(V) adsorption on the amino-MIL-68(Al) is an endothermic process^79^. In other words, higher temperatures accelerate mass-transfer and process kinetics, resulting in an improved adsorption process. Also, a positive value of ΔS° means that chaos increases at the solid–liquid interface. In such a situation ion exchange occurs during As(V) uptake^80^.

**Table 6:** Thermodynamic parameters of As(V) adsorption on amino-MIL-68(Al).

T (K)	ΔG° (KJ/mol)	ΔH° (KJ/mol)	ΔS° (J/(mol. K))
298	− 4.076	70.718	0.251
303	− 5.370		
308	− 6.874		
313	− 8.194		
318	− 8.895		
323	− 10.478		

### Comparison of efficiency and reusability of amino-MIL-68(Al) and MIL-68(Al) for As(V) removal

**Figure 9:**
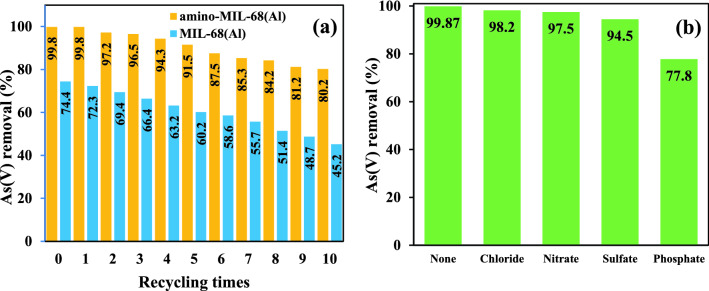
As(V) removal efficiency by amino-MIL-68(Al) and MIL-68(Al) for ten consecutive reuse cycles under optimal conditions (**a**). Interventional effect of anions on As(V) removal efficiency by amino-MIL-68(Al) (The oncentration of anions = 1 mM) (**b**).

The results of Sect. “Characterization of amino-MIL-68(Al) and the effect of amine group in improving MOF properties” showed that the functionalization of MIL-68(Al) with the amine group (-NH_2_) was able to significantly improve the porosity and surface area of the MOF. Therefore, to confirm the effect of the amine group in improving the absorption of As(V), the removal efficiency and reusability of amino-MIL-68(Al) and MIL-68(Al) were compared for ten consecutive reuse cycles under optimal conditions. MOF regeneration was performed after each reuse round using 0.01 M nitric acid solution^65,75^. As shown in Fig. 9a, the removal efficiency of As(V) with amino-MIL-68(Al) is significantly higher compared to MIL-68(Al). Accordingly, in the first round of use, the removal efficiency of As(V) with amino-MIL-68(Al) and MIL-68(Al) was 99.8% and 74.4%, respectively. The Functionalization of MOFs with the amine group not only increases the number of electron-rich nitrogen sites and the positive charge in the MOF structure but also strengthens hydrogen bonds and increases the adsorption rate^46^. In this regard, Haque et al., reported that NH_2_-MIL-101(Al) has a higher adsorption capacity to remove methylene blue than MIL-101(Al)^81^. In addition, the results showed that amino-MIL-68(Al) has more reusability compared to MIL-68(Al) so that the removal efficiency of As(V) with amino-MIL-68(Al) and MIL-68(Al) decreased by about 19.6% and 29.2% after ten reuse cycles, respectively. These results confirm that functionalization of MIL-68 (Al) with the amine group not only increases the surface area of MOF but also improves As(V) removal efficiency and MOF reusability.

### Effect of interfering anions on As(V) removal efficiency

Natural waters and industrial wastewater are always contaminated with a variety of ion-interfering species that dramatically affect the efficiency of the adsorption process. In this study, the effect of chloride, nitrate, sulfate, and phosphate anions at a constant concentration of 1 mM and under optimal laboratory conditions (pH = 3, MOF dose = 0.4 g/L, reaction time = 80 min and As(V) concentration = 2.5 mg/L) was investigated on the efficiency of As(V) removal, the results of which are presented in Fig. 9b. As can be seen, the presence of chloride, nitrate, and sulfate anions in the samples solution does not have much interference with As(V) adsorption on MOF. However, in the presence of phosphate, a significant reduction is observed in the As(V) removal efficiency. Phosphate competes with As(V) for active sites at the MOF surface, resulting in inhibition of As(V) adsorption. The high intervening effect of phosphate with As(V) adsorption can be attributed to similar physicochemical properties of these two elements^60^.

## Conclusion

In the present study, amino-MIL-68(Al) was prepared by solvothermal method using 2-aminoterephthalic acid as a ligand. FESEM, XRD, FTIR and EDX-mapping analysis confirmed the synthesis of MOF structures. The results of N_2_ adsorption/desorption isotherm data showed that the BET surface area of the synthesized MOF is > 1000 m^2^/g. Experimental As(V) removal efficiency under optimal conditions (pH = 3, MOF dose = 0.4 g/L, reaction time = 80 min and As(V) concetration = 2.5 mg/L) was obtained 99.87%. Experimental data were fitted with the nonlinear form of isothermand kinetic models. The results showed that the adsorption of As(V) fits well with the Langmuir model. Accordingly, the maximum As(V) adsorption capacity was obtained 74.29 mg/g. The fit of the data with the pseudo-second-order kinetic model showed that the mechanism of As(V) adsorption has a chemical nature. In addition, thermodynamic studies revealed that As(V) adsorption is a spontaneous endothermic process. Based on the data of the present study, MOF is a promising and recyclable adsorbent for the removal of As(V) from contaminated water.
